# CTLA-4 blockade boosts the expansion of tumor-reactive CD8^+^ tumor-infiltrating lymphocytes in ovarian cancer

**DOI:** 10.1038/s41598-020-60738-4

**Published:** 2020-03-03

**Authors:** Christina Friese, Katja Harbst, Troels Holz Borch, Marie Christine Wulff Westergaard, Magnus Pedersen, Anders Kverneland, Göran Jönsson, Marco Donia, Inge Marie Svane, Özcan Met

**Affiliations:** 1 Center for Cancer Immune Therapy (CCIT), Department of Oncology, Herlev Hospital, Herlev, Denmark; 2 Department of Clinical Sciences Lund, Division of Oncology and Pathology, Lund University, Lund, Sweden; 3 Department of Immunology and Microbiology, Faculty of Health and Medical Sciences, University of Copenhagen, Copenhagen, Denmark

**Keywords:** Ovarian cancer, Immunotherapy, Translational research, Tumour immunology

## Abstract

Adoptive cell therapy (ACT) with autologous tumor-infiltrating lymphocytes (TILs) can induce durable complete tumor regression in patients with advanced melanoma. Efforts are currently underway to expand this treatment modality to other cancer types. In the microenvironment of ovarian cancer, the engagement of co-inhibitory immune checkpoint molecules such as CTLA-4 can lead to the inactivation of TILs. Thus, approaches that directly manipulate co-inhibitory pathways within the tumor microenvironment might improve the expansion of tumor-reactive TILs. The initial expansion of TILs for ACT from tumor fragments provides a window of opportunity to manipulate an intact tumor microenvironment and improve CD8^+^ T-cell output and TIL tumor reactivity. To exploit this, we used a CTLA-4-blocking antibody, added during the initial TIL culture, and found that the blockade of CTLA-4 favored the propagation of CD8^+^ TILs from ovarian tumor fragments. Interestingly, adding the CTLA-4 blocking antibody in the initial phase of the TIL culture resulted in more potent anti-tumor TILs in comparison to standard TIL cultures. This phenotype was preserved during the rapid expansion phase. Thus, targeting CTLA-4 within the intact tumor microenvironment of tumor fragments enriches tumor-reactive TILs and may improve clinical outcome of TIL-based ACT in ovarian cancer.

## Introduction

Ovarian cancer is the eighth most common cause of cancer death in women worldwide^1^. Ovarian cancer is often diagnosed at an advanced stage (e.g. International Federation of Gynecology and Obstetrics (FIGO) stage III or IV)^2,3^ and often characterized by a high tumor burden at diagnosis^2^. The primary treatments consist of platinum-based chemotherapy and surgery^3^, but the majority of patients with ovarian cancer relapse after the initial treatment^3^. The prognosis of advanced ovarian cancer is very poor, with a 5-year survival of 39% and 17% in stage III and IV, respectively^4^. Thus, more efficient treatment modalities are highly warranted.

Checkpoint inhibitors have demonstrated clinical benefit in a small proportion of patients with ovarian cancer. Responses to checkpoint inhibitors have been reported consistently at rates below 15%^5–9^.

Several studies have previously demonstrated a positive prognostic value of tumor-infiltrating lymphocytes (TIL) in ovarian cancer. The presence of intraepithelial CD8^+^ cytotoxic TILs is associated with improved survival^10–12^. On the other hand, infiltration with regulatory T cells (Tregs), which can inhibit cytotoxic T cells, is associated with a poor prognosis^13^. Additionally, Sato *et al*. demonstrated that a high CD8^+^/Tregs ratio is associated with improved survival^12^.

Adoptive T-cell therapy (ACT) based on autologous TILs takes advantage of tumor-reactive T cells naturally present in the tumor lesions^14^ and has shown overall response rates of around 50% with durable complete responses in 15–20% of the patients in metastatic melanoma^15–20^. In addition, clinical responses in other epithelial cancers with ACT were recently reported by the NCI^21^. Clinical studies using ACT in ovarian cancer have demonstrated contradicting results^22–24^. A preclinical study and a phase I study treating six patients with metastatic ovarian cancer with ACT completed at our center have shown that ACT in metastatic ovarian cancer is feasible, but clinical responses were transient^25,26^. A phase I/II study combining ACT with checkpoint inhibitors has just been finalized (Kverneland *et al*., manuscript in preparation).

The studies mentioned above show that tumor-reactive T cells are present in ovarian cancer lesions, but the limited clinical effect suggests that they are possibly inhibited. The activity of TILs can be impeded by antigen-presenting cells or tumor cells through immune checkpoints such as programmed cell death protein 1 (PD-1) or cytotoxic T-lymphocyte-associated protein 4 (CTLA-4) expressed on the T-cell surface, which can be blocked by checkpoint inhibitors such as ipilimumab (anti-CTLA-4), nivolumab and pembrolizumab (both anti-PD-1)^27^.

In previous TIL trials in metastatic melanoma, a high number of infused TILs and a large proportion of CD8^+^ T cells in the infusion product have been associated with better clinical outcome^18^. Also, TIL reactivity towards autologous tumor cell lines *in vitro* has been associated with clinical outcome^28^. In this study, we hypothesized that directly manipulating co-inhibitory pathways within the initial tumor fragment cultures for ACT might improve the expansion of tumor-reactive TILs from ovarian tumor fragments. In order to block the co-inhibitory pathway, a CTLA-4-targeting antibody was added during the TIL expansion. Subsequently, the phenotype and functionality were analyzed by flow cytometry and the T-cell clonality was analyzed by T-cell receptor (TCR) sequencing.

## Results

### Demographics of patient samples

Ovarian cancer metastatic lesions were collected from 14 ovarian cancer patients that underwent surgery between 2014 and 2018 with an ECOG performance status 0–1. The patients were primarily diagnosed with ovarian cancer FIGO stage IIIc-IV between 2010 and 2017. The median time from diagnosis to tumor removal was three years and the patients had received a median of four lines of therapy at that time.

The samples were attained from three different clinical protocols performed at our center. One patient (EOC.TIL.27) had a tumor removed as part of the standard care, while the others underwent tumor removal as part of a clinical trial with adoptive cell therapy (GY1508 and GY1721 - NCT02482090 and NCT03287674). The latest cohort (GY1721) also received one dose of ipilimumab (3 mg/kg) two weeks prior to tumor removal as part of the clinical trial.

### Expansion and phenotype of ovarian cancer TILs

**Figure 1:**
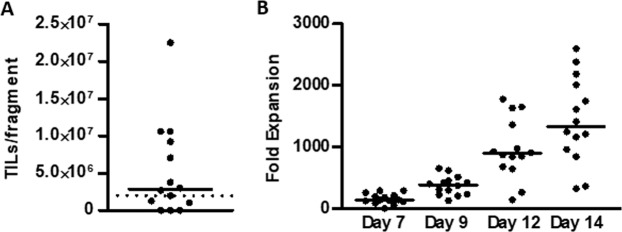
Expansion of ovarian cancer TILs. TIL cultures were established from tumor fragments and subsequently rapidly expanded over a course of 14 days. **(A)** Scatter plot showing the initial TIL yield per fragment. Data are presented with median. **(B)** Scatter plot showing the fold expansion during the rapid expansion. Data are presented with median.

Resected tumor lesions were cut into 1 to 3 mm^3^ small fragments. One fragment was plated per well and cultured in media containing 6,000 IU/ml IL-2. TIL cultures could be established in 9 of 14 patients (64%) as shown in Fig. 1A. Median days in culture were 29 (range [16–36] days) with a median number of 2.87 × 10^6^ (range [0–22.5 × 10^6^]) TILs recovered per fragment. Next, we evaluated whether TILs could be expanded to clinically relevant numbers using a two-week rapid expansion protocol (REP)^29^. If TIL cultures could not be established, TILs previously established in the clinical studies (Trial Number NCT02482090 and NCT03287674) were used. The median fold expansion was 1,300 (range [323–2,594]) (Fig. 1B).

**Figure 2:**
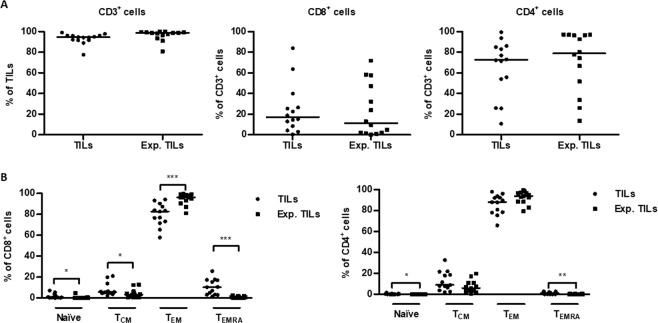
Phenotype of ovarian cancer TILs. **(A)** Scatter plots showing the proportions of CD3^+^, CD8^+^ and CD4^+^ cells in initial TIL cultures (TILs) and expanded TIL cultures (Exp. TILs). Data are presented with median. **(B)** Scatter plots showing the distribution of naïve (T_N_), central memory (T_CM_), effector memory (T_EM_) and effector memory cells re-expressing CD45RA (T_EMRA_) cells within CD3^+^ CD8^+^ and CD3^+^ CD4^+^ T cells in initial TIL cultures (TILs) and expanded TIL cultures (Exp. TILs). T_N_, T_CM_, T_EM_ and T_EMRA_ cells were defined as CD45RA^+^ CD62L^+^, CD45RA^−^ CD62L^+^, CD45RA^−^ CD62L^−^ and CD45RA^+^ CD62L^−^, respectively. Data are presented with median. Initial TILs and expanded TILs were compared using Wilcoxon signed rank test. Statistically significant differences are indicated with *, ** or *** for p-values less than 0.05, 0.01 or 0.001, respectively.

Subsequently, we determined the phenotype of the TIL cultures before and after expansion using flow cytometry. As shown in Fig. 2A, the majority of cells in both TIL cultures expressed CD3 and CD4. There was no difference in the distribution of CD3^+^, CD8^+^ and CD4^+^ T cells in TIL cultures before and after expansion. We also determined the memory phenotype of CD4^+^ and CD8^+^ T cells. Naïve (T_N_), central memory (T_CM_), effector memory (T_EM_) and effector memory cells re-expressing CD45RA (T_EMRA_) were defined as CD45RA^+^ CD62L^+^, CD45RA^−^ CD62L^+^, CD45RA^−^ CD62L^−^ and CD45RA^+^ CD62L^−^, respectively. The majority of CD4^+^ and CD8^+^ T cells in initial TIL cultures were T_EM_ (Fig. 2B). In CD8^+^ T cells, the fraction of T_EM_ increased significantly after the expansion (p-value = 0.0007), while the fraction of T_N_, T_CM_ and T_EMRA_ decreased significantly (p-values = 0.03, 0.05 and 0.0002). In CD4^+^ cells, T_N_ and T_EMRA_ fractions decreased significantly after the rapid expansion (p-values = 0.01 and 0.008). These results suggest that TILs from patients with ovarian cancer expanded to clinically relevant numbers mainly consist of CD3^+^ CD4^+^ T cells with a T_EM_ phenotype.

### Expansion of TILs after the addition of anti-CTLA-4 antibody

**Figure 3:**
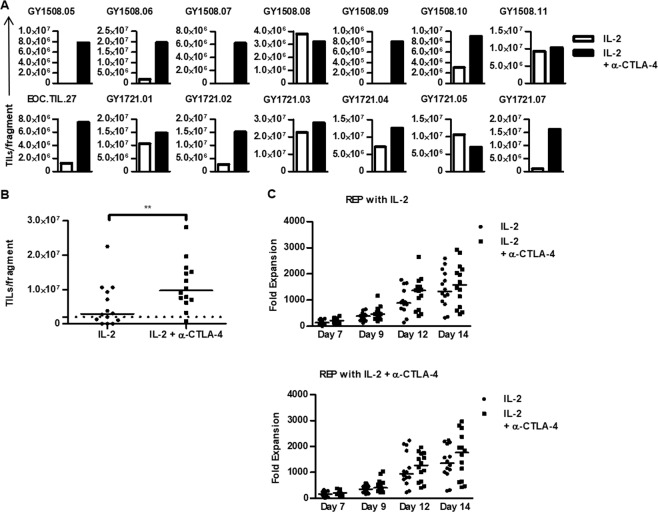
Expansion of ovarian cancer TILs with anti-CTLA-4. TIL cultures were established from tumor fragments and subsequently rapidly expanded over a course of 14 days. For each patient, two initial and four expanded TIL cultures were established. Both the initial TIL culture and the rapid expansion were performed with high-dose IL-2 alone or supplemented with anti-CTLA-4 antibody. **(A)** Bar charts illustrating the TIL yield per fragment of the initial TIL cultures for each patient. **(B)** Scatter plot showing the TIL yield per fragment of the initial TIL culture under the two culture conditions. The dotted line indicates the threshold for a successful TIL culture. Data are presented with median. **(C)** Scatter plots showing the fold expansion during the rapid expansion with high dose IL-2 alone (upper figure) or additionally supplemented with anti-CTLA-4 antibody. The legend indicates the culture conditions during the initial TIL expansion. Data are presented with median. The culture conditions were compared using Wilcoxon signed rank test. Statistically significant differences are indicated with *, ** or *** for p-values less than 0.05, 0.01 or 0.001, respectively.

We hypothesized that ovarian cancer TILs might be inactivated through co-inhibitory interactions such as the immune checkpoint CTLA-4. Thus, we tested the effect of blocking this interaction by adding anti-CTLA-4 antibody to the TIL cultures. CTLA-4 blockade promoted TIL growth from tumor fragments of 13 out of 14 (93%) patients compared to TIL growth from 9 out of 14 (64%) patients when tumor fragments were cultured in standard IL-2 conditions. Median culture time was 29 days (range [16–36] days) for both culture conditions with a significantly increased number of TILs recovered from TIL cultures supplemented with anti-CTLA-4 with a median of 9.65 × 10^6^ cells per fragment (range [0.8–28.1 × 10^6^], Fig. 3A,B) compared to a median number of 2.87 × 10^6^ (range [0–22.5 × 10^6]^]) for TIL cultures (p-value = 0.001) with only IL-2. The outgrowth of TILs from tumor fragments was higher in 12 of 14 patients when cultured with anti-CTLA-4 antibody. Interestingly, the increase in TIL growth was more profound when using tumor fragments from patients not pre-treated with anti-CTLA-4 antibody before TIL therapy compared to those pre-treated (Table 1, Trial Number NCT02482090 and NCT03287674).

**Table 1:** Expansion of TILs from Ovarian Cancer Tumors.

Sample ID	Previous anti-CTLA-4 treatment	Condition	Number of fragments	Total TIL number (×10^6^)	TILs per fragment (×10^6^)	Expansion time (Days)
GY1508.05	No	IL-2	4	0.1	0.03	35
		IL-2 + anti-CTLA-4	4	31.2	7.8	35
GY1508.06	No	IL-2	10	20	2	30
		IL-2 + anti-CTLA-4	10	196	19.6	28
GY1508.07	No	IL-2	6	0	0	32
		IL-2 + anti-CTLA-4	6	37	6.17	32
GY1508.08	No	IL-2	8	38	4.75	33
		IL-2 + anti-CTLA-4	8	16.1	2.01	33
GY1508.09	No	IL-2	8	0	0	25
		IL-2 + anti-CTLA-4	8	6.4	0.80	32
GY1508.10	No	IL-2	8	24.2	3.03	28
		IL-2 + anti-CTLA-4	8	72.4	9.05	26
GY1508.11	No	IL-2	8	74	9.25	26
		IL-2 + anti-CTLA-4	4	41	10.25	26
EOC.TIL.27	No	IL-2	12	15.4	1.28	35
		IL-2 + anti-CTLA-4	12	90	7.50	35
GY1721.01	Yes	IL-2	8	84.9	10.61	18
		IL-2 + anti-CTLA-4	8	117.6	14.70	18
GY1721.02	Yes	IL-2	8	21.7	2.71	32
		IL-2 + anti-CTLA-4	8	120.7	15.09	30
GY1721.03	Yes	IL-2	8	180.2	22.53	21
		IL-2 + anti-CTLA-4	8	224.9	28.11	21
GY1721.04	Yes	IL-2	8	56.7	7.09	25
		IL-2 + anti-CTLA-4	8	100.2	12.53	22
GY1721.05	Yes	IL-2	8	84.5	10.56	16
		IL-2 + anti-CTLA-4	8	56.2	7.03	16
GY1721.07	Yes	IL-2	4	4.2	1.04	36
		IL-2 + anti-CTLA-4	6	97.9	16.32	36

To test the effect of CTLA-4 blockade during the rapid expansion, each TIL culture was expanded with either IL-2 in a standard REP or by additionally adding anti-CTLA-4 antibody on day 0 and day 7 during the REP, thereby generating expanded TILs in four conditions. The median fold expansion in all conditions was higher than 1,300-fold (Fig. 3C). Adding CTLA-4 antibody during the REP did not affect the fold expansion of TILs. However, TILs subjected to CTLA-4 blockade during tumor fragment cultures expanded to greater levels compared to TILs from tumor fragment cultures in IL-2 only (median 1,600 and 1,800-fold vs. 1,300 and 1,400-fold). Thus, CTLA-4 blockade of TILs during the early expansion phase improves the total numbers of TILs derived from ovarian tumor fragments.

### Phenotype of TILs after CTLA-4 blockade

**Figure 4:**
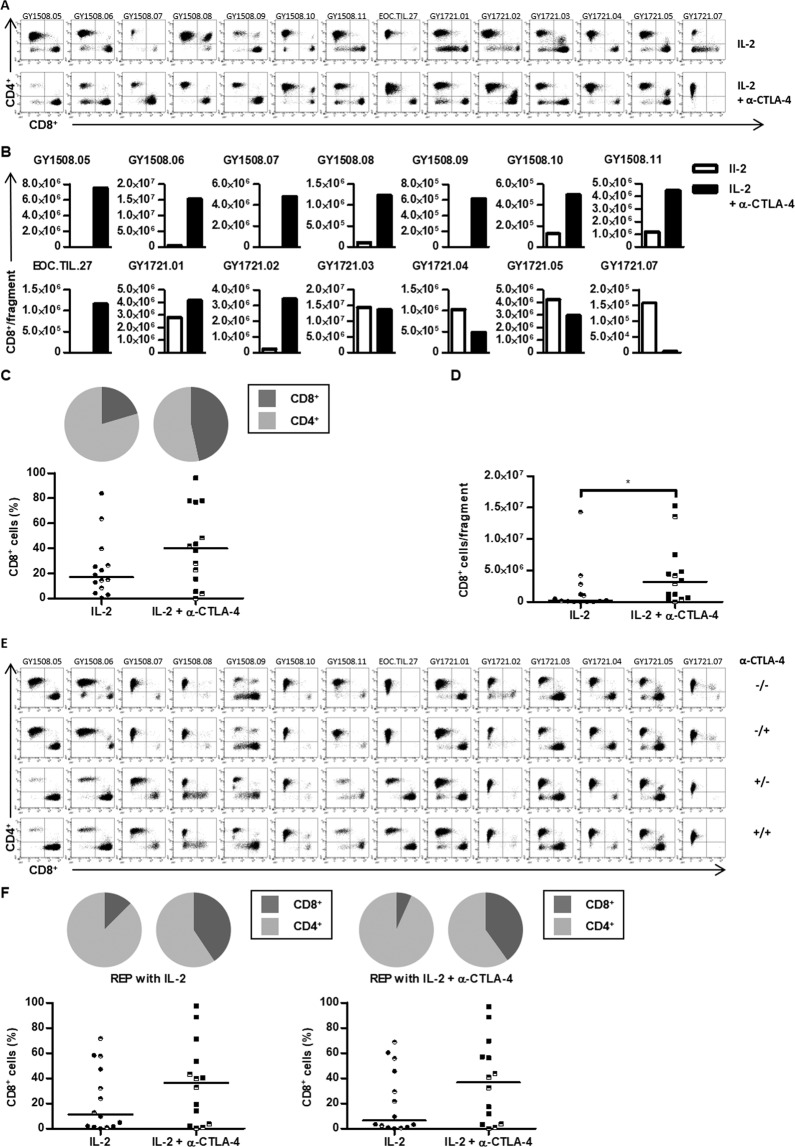
T-cell subsets of ovarian cancer TILs with anti-CTLA-4. **(A)** FACS dot plots showing the distribution of CD4^+^ and CD8^+^ T cells within the CD3^+^ T cell population in the initial TIL cultures for each patient. **(B)** Bar charts showing the yield of CD8^+^ T cells per fragment in the initial TIL cultures for each patient. **(C)** Scatter plot showing the CD3^+^CD8^+^ T cell frequency of the initial TIL cultures. Pie charts showing the median distribution of CD4^+^ and CD8^+^ T cells within the CD3^+^ T cell population. **(D)** Scatter plot showing the CD3^+^CD8^+^ T cell yield in the initial TIL cultures. **(E)** FACS dot plots showing the distribution of CD4^+^ and CD8^+^ T cells within the CD3^+^ T cell population in the expanded TIL cultures for each patient. The + and − indicate if anti-CTLA-4 antibody was added to the cultures; before the slash indicates supplementation during the initial TIL culture and after the slash indicates supplementation during the rapid expansion. **(F)** Scatter plots showing the CD3^+^CD8^+^ T cell frequency of the TIL cultures expanded with high-dose IL-2 alone (left figure) or supplemented with anti-CTLA-4 antibody (right figure). The legend indicates the culture conditions during the initial TIL expansion. Pie charts showing the distribution of CD4^+^ and CD8^+^ T cells within the CD3^+^ T cell population. **(C**,**D**,**F**) The patients included in this study were anti-CTLA-4 naïve (closed symbol) or pre-treated with anti-CTLA-4 (half-closed symbol). Data are presented with median. Initial TILs and expanded TILs were compared using Wilcoxon signed rank test. Statistically significant differences are indicated with *, ** or *** for p-values less than 0.05, 0.01 or 0.001, respectively.

Next, we evaluated the effect of CTLA-blockade on the phenotype of TILs during initial TIL growth and after expansion. Flow cytometric analysis showed no difference in the number of CD3^+^ T cells before or after cell expansion irrespective of CTLA-4 blockade (Supplementary Fig. S1). In contrast, the proportion of CD8^+^ T cells was increased in TIL cultures when anti-CTLA-4 antibody was added to the tumor fragments during the initial TIL growth compared to TIL outgrowth when cultured only with IL-2 (Fig. 4A–C and Supplementary Table S1). In addition, the proportion of CD4^+^ cells was decreased in a corresponding manner (Fig. 4A–C and Supplementary Table S2). Also, a significantly higher number of CD8^+^ T cells were recovered per fragment after CTLA-4 blockade (Fig. 4D). Importantly, the proportion of CD8^+^ T cells recovered from tumor fragments after CTLA-4 blockade was sustained when TIL cultures were rapidly expanded (Fig. 4E,F). Thus, the addition of anti-CTLA-4 antibody during the initial TIL culture increases the proportion of CD8^+^ T cells in ovarian cancer TILs and leads to an increased amount of CD8^+^ T cells after the rapid expansion without additional anti-CTLA-4 antibody during the rapid expansion.

The effect of CTLA-4 blockade on the outgrowth of T-cell subsets was further examined using flow cytometry to determine the expression of activation and co-stimulatory markers, and the expression of exhaustion markers. A majority of CD8^+^ and CD4^+^ T cells in initial TIL cultures with or without CTLA-4 blockade had a T_EM_ phenotype (Supplementary Fig. S2). In CD4^+^ T cells, the proportion of T_EMRA_ cells was reduced in TILs supplemented with anti-CTLA-4 antibody compared to TILs cultured with IL-2 alone (Supplementary Fig. S2). There was no difference in any of the activation and co-stimulatory markers CD27, CD28, CD56, CD69, CD137 and the exhaustion markers LAG-3, BTLA, TIM-3, PD-1 and CD57 in the initial TIL cultures (Supplementary Fig. S2). Furthermore, there was no difference in the expression of ICOS or CTLA-4 in the initial TIL cultures (Supplementary Fig. S2).

In the expanded TILs, the majority of CD8^+^ and CD4^+^ T cells were T_EM_ and the expression of most activation, co-stimulatory and exhaustion markers was comparable (Supplementary Fig. S3). Only the expression of CD69 in CD4^+^ T cells was lower in TILs expanded from anti-CTLA-4 supplemented TILs compared to those expanded from non-supplemented TILs (p-value = 0.04). The expression of ICOS was lower on CD8^+^ and CD4^+^ T cells in TILs expanded from initial TIL cultures supplemented with anti-CTLA-4 antibody compared to those expanded from non-supplemented TIL cultures (Supplementary Fig. S3, p-values = 0.02 and 0.15). These results indicate that the addition of anti-CTLA-4 antibody during the initial TIL expansion does not induce major changes in the memory phenotype, the activation or the exhaustion state of expanded TILs. Moreover, there were no differences in the memory phenotype, the expression of activation and co-stimulatory markers and the expression of exhaustion markers when anti-CTLA-4 antibody was added during the rapid expansion (Supplementary Figs. S4 and S5).

### Reactivity of TILs to autologous tumors

Next, we tested whether TIL cultures were able to recognize autologous tumor cell lines. In four of the fourteen patients included, tumor cell lines could be established, and the tumor reactivity of the TILs was tested. We co-cultured TILs and autologous tumor cell lines for five hours in the presence of Golgi-Plug and measured the expression of CD107a and the intracellular accumulation of TNF and IFN-γ. Tumor-reactive T cells were defined as cells expressing CD107a or secreting one of the cytokines^30^.

**Figure 5:**
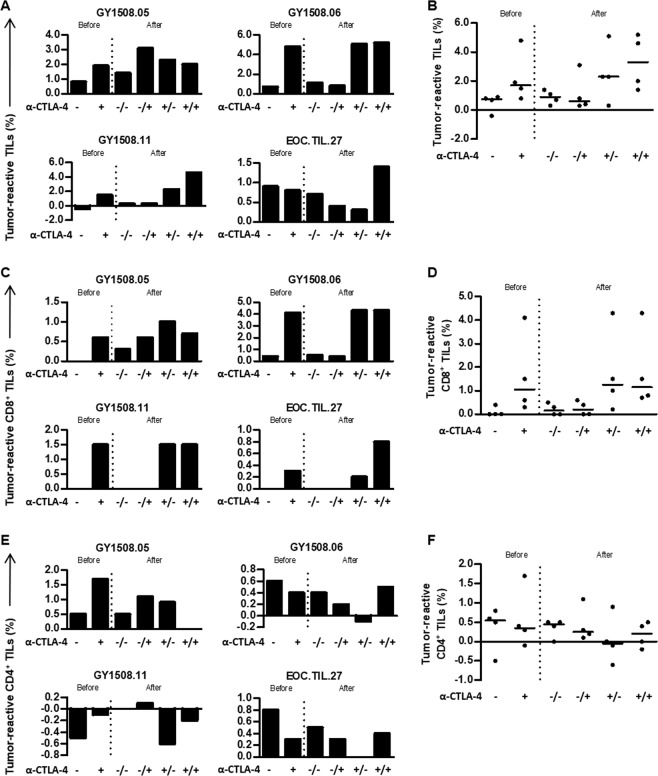
*In vitro* tumor reactivity of ovarian cancer TILs with anti-CTLA-4. The tumor reactivity of the *in vitro* expanded TILs was evaluated by defining the frequency of TILs expressing at least one of the following T-cell functions: TNF, IFN-γ or CD107a upon stimulation with autologous tumor cell lines treated with low-dose IFN-γ (100 IU/ml). The frequency of tumor-reactive cells in an unstimulated sample was subtracted from the stimulated samples. Initial TIL cultures (before) were established using high-dose IL-2 or supplemented with anti-CTLA-4. Subsequently, both TIL cultures were expanded in a rapid expansion (after) under two different conditions, supplemented with anti-CTLA-4 antibody or not supplemented. The + and - indicate if anti-CTLA-4 antibody was added to the cultures; for the expanded TIL cultures, before the slash indicates supplementation during the initial TIL culture and after the slash indicates supplementation during the rapid expansion. Bar charts **(A**,**C**,**E)** and scatter plots **(B**,**D**,**F**) showing the tumor-reactive TILs **(A**,**B)**, CD8^+^ cells **(C**,**D)** and CD4^+^ cells **(E**,**F)** for each patient **(A,C**,**E)** or for all patients tested **(B,D**,**F**, n = 4**)**. Data are presented with median.

Reactivity against autologous tumor cell lines was detected in three out of four patient TIL cultures before expansion, while all rapidly expanded TILs were able to recognize the autologous tumor cell line (Fig. 5A). The median proportion of tumor-reactive T cells was 0.75% in initial TILs and 0.9% in expanded TIL cultures (Fig. 5B). Tumor-reactive CD8^+^ T cells could be detected in pre-expanded TIL cultures from one out of four patients, and in two out of four TIL cultures after expansion (Fig. 5C,D). For CD4^+^ T cells, tumor reactivity was detected in three out of four TIL cultures, both before and after cell expansion (Fig. 5E,F).

**Figure 6:**
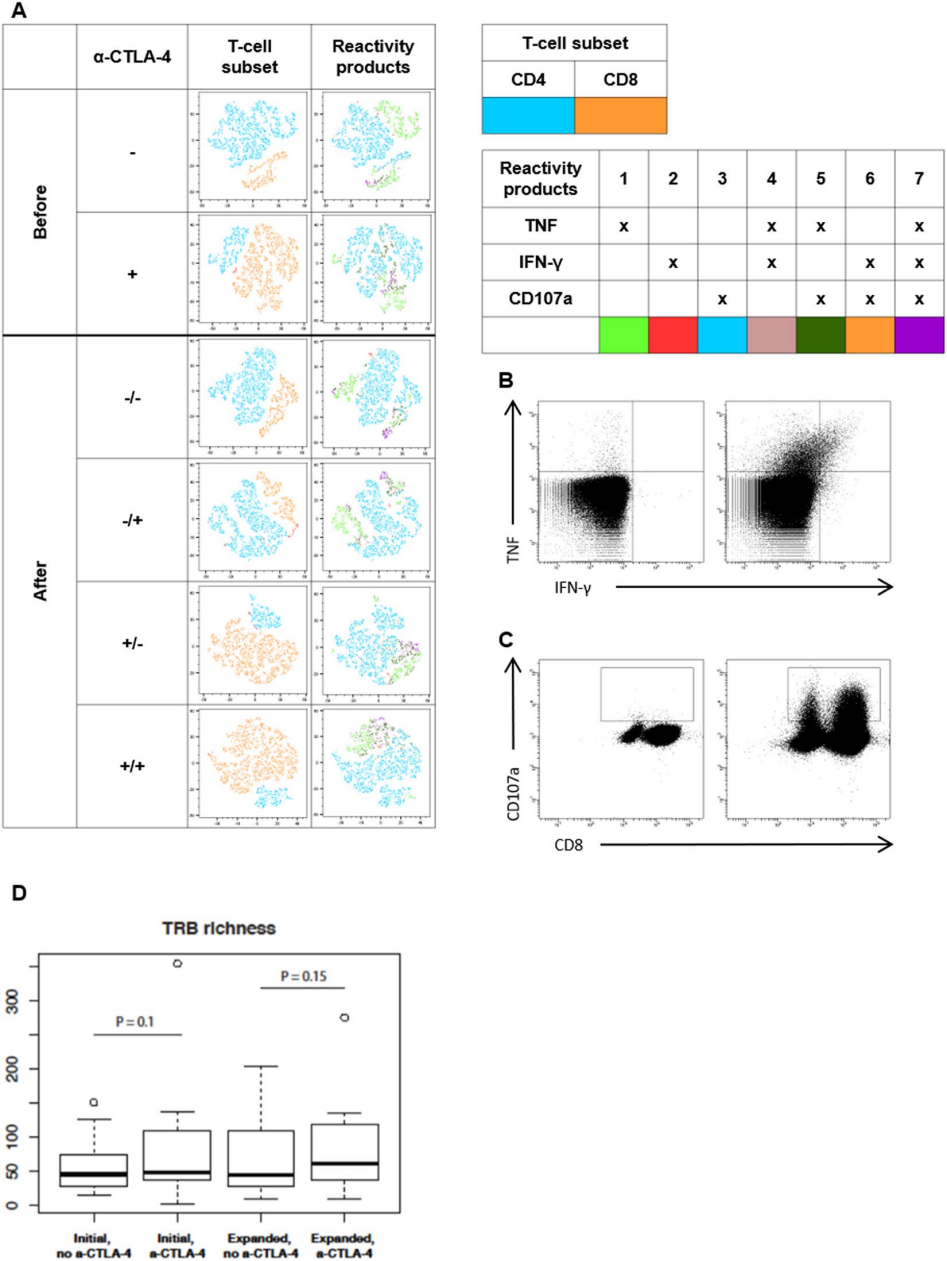
Reactivity products and TRB richness of ovarian cancer TILs with anti-CTLA-4. The tumor reactivity of the initial and expanded TILs was evaluated by measuring the secretion of TNF and IFN-γ or the expression of CD107a upon stimulation with autologous tumor cell lines treated with low-dose IFN-γ (100 IU/ml). Initial TIL cultures were established using high-dose IL-2 or supplemented with anti-CTLA-4. Subsequently, both TIL cultures were expanded in a rapid expansion under two different conditions, supplemented with anti-CTLA-4 antibody or not supplemented. **(A)** tSNE plots showing the distribution of reactivity products of TILs in initial (before) and expanded TIL cultures (after). The + and - indicate if anti-CTLA-4 antibody was added to the cultures; for the expanded TIL cultures, before the slash indicates supplementation during the initial TIL culture and after the slash indicates supplementation during the rapid expansion. **(B)** FACS plots showing cytokine production from TILs alone (negative control) and TILs stimulated with an autologous tumor cell line treated with low –dose IFN-γ from a representative patient. **(C)** FACS plots showing CD107a mobilization of TILs alone (negative control) and TILs stimulated with an autologous tumor cell line treated with low-dose IFN-γ. **(D)** TRB richness in the initial and expanded cultures with and without anti-CTLA-4 antibody, as derived by TCR repertoire analysis.

We next tested whether the tumor-reactive potential of TILs was affected by the addition of anti-CTLA-4 antibody during TIL culture. As shown in Fig. 5, the provision of CTLA-4 blockade to the tumor fragments yielded a higher number of tumor-reactive T cells before and after rapid expansion. The majority of the reactive cells were CD8^+^ T cells (Fig. 5C,D). The addition of anti-CTLA-4 antibody during the rapid expansion did not change the tumor reactivity of TILs and CD8^+^ T cells. Reactivity of CD4^+^ T cells was generally low compared to CD8^+^ T-cell tumor reactivity (Fig. 5E,F). The majority of tumor-reactive TILs expressed CD107a or secreted TNF (Fig. 6A). An increased proportion of CD8^+^ T cells in the initial TILs supplemented with anti-CTLA-4 antibody compared to non-supplemented TILs secreted TNF and IFN-γ and expressed CD107a upon co-culture with autologous tumor cell lines (Fig. 6A). This phenomenon is preserved in expanded TILs independent of the addition of anti-CTLA-4 during the rapid expansion. Cytokine production (TNF and IFN-γ) and CD107a mobilization in CD8^+^ T cells in expanded TILs of one representative patient are shown in Fig. 6B,C. Overall, these experiments demonstrate that the addition of anti-CTLA-4 antibody in the tumor fragment cultures can improve the expansion of tumor-reactive TILs.

### TCR repertoire analysis

In order to investigate whether the addition of anti-CTLA-4 antibody during TIL expansion remodels TCR repertoire, we performed TCR repertoire analysis on initial TIL cultures supplemented with or without anti-CTLA-4 antibody and on REP cultures expanded from these without the addition of anti-CTLA-4 antibody from 12 patients. The addition of anti-CTLA-4 antibody to TIL cultures did not result in a change in TRB (gene encoding the TCR β chain) diversity or evenness (Supplementary Fig. S6). However, although not significant, there was an increase in TRB richness in initial or expanded cultures when anti-CTLA-4 antibody was added (Fig. 6D).

## Discussion

Given the potential that TIL therapy holds for the treatment of metastatic disease, identifying methodologies to enrich and expand tumor-reactive TILs may improve clinical outcomes of TIL-based ACT in ovarian cancer. In this study, we have shown that the addition of anti-CTLA-4 antibody to tumor fragments from metastatic ovarian cancer does not only promote the outgrowth of TILs but also favors the propagation of CD8^+^ T cells and thus enhances the anti-tumor reactivity of TILs. These findings are important in overcoming the challenges of TIL-based ACT in recurrent ovarian cancer.

In metastatic melanoma, a high number of infused TILs, a high proportion of CD8^+^ T cells and TIL reactivity towards autologous tumor cell lines *in vitro* have previously been associated with improved clinical outcome^18,28^. In earlier TIL trials in ovarian cancer, response rates have generally been lower compared to metastatic melanoma^16,22,24,31^. Possible explanations could be lower numbers of infused TILs, lower proportions of CD8^+^ T cells in the infusion product and lower TIL reactivity. The latter could in turn be due to a lower mutational burden in other types of cancer compared to melanoma^32^. However, induction of durable clinical responses with TIL therapy is possible in other cancers than melanoma, if the expansion method favors the reactive T-cell clones^21,33^.

In this study, we show increased TIL proliferation, increased proportion of CD8^+^ T cells and increased TIL reactivity towards autologous tumor cell lines *in vitro* when CTLA-4 blockade was introduced during the initial TIL expansion from ovarian tumor fragments. TIL cultures subjected to CTLA-4 blockade did also expand into greater numbers after rapid expansion when compared to standard IL-2 culture conditions.

In line with these results, a recent clinical study conducted at our center did also show increased TIL proliferation and *in-vitro* tumor reactivity when patients with ovarian cancer were pre-treated with one dose of ipilimumab before resection of tumor material for preparation of ACT (Kverneland *et al*., manuscript in preparation). Our results also corroborate findings in another study at our center that has shown more frequent T-cell responses against common tumor antigens in patients treated with ipilimumab as compared to anti-CTLA-4 naïve patients with metastatic melanoma^34^. Another study demonstrated that blockade of CTLA-4 and PD-1 increased proliferation of murine colon carcinoma and ovarian carcinoma TILs expressing these immune checkpoints^35^. In melanoma, triple blockade of BTLA-4, TIM-3 and PD-1 was shown to enhance the expansion and proliferation of specific CD8^+^ PBMCs^36^.

Fewer CD4^+^ TILs expanded from initial TIL cultures supplemented with anti-CTLA-4 expressed CD69 compared to those expanded without anti-CTLA-4. Han *et al*. have shown a regulatory function of CD69 on Tregs in mice^37^. Thus, a reduced expression of CD69 by CD4^+^ T cells could indicate a reduced suppressive activity and could allow an increased cytotoxicity of CD8^+^ cytotoxic T cells. Moreover, suppression by Tregs seems to be mediated by the inhibition of the IL-2 receptor anti-chain induced by CTLA-4 and membrane-bound TGF-β1^37^. This interaction is blocked in the *in-vitro* TIL cultures by anti-CTLA-4. CD69^+^ Tregs have been shown to express higher levels of suppressive markers, such as ICOS^38^. An increased expression of ICOS has previously been shown in Tregs in TILs compared to peripheral Tregs. It was associated with an increased expression of Treg markers, the secretion of IL-10 and a stronger suppressive activity^39–42^. Importantly, we found that ICOS expression was reduced on both CD4^+^ and CD8^+^ T cells in TILs expanded from initial TIL cultures supplemented with anti-CTLA-4 antibody. We determined the frequency of Tregs in the patients with a profound increase of CD8^+^ cells when the initial TIL culture was treated with anti-CTLA-4 antibody using flow cytometry. The proportion of Tregs (CD4^+^CD25^+^CD127^low^FoxP3^+^) within the CD3^+^ compartment was reduced in 3 out of 4 patients (data not shown). Further, a recent study comparing ovarian cancer-Tregs with melanoma-Tregs demonstrated an increased activation state and a more suppressive effect of ovarian cancer-Tregs on CD8^+^ T cells compared to melanoma-Tregs^43^. This could explain the effect of anti-CTLA-4 antibody on the ovarian cancer TIL cultures, but no effect on melanoma TIL cultures (data not shown). Another study conducted at our center showed a reduced Treg infiltration of the TME when patients with ovarian cancer were treated with one dose of ipilimumab before resection of tumor material for preparation of ACT with autologous TILs compared to patients that were not pre-treated with ipilimumab (Westergaard *et al*., manuscript in preparation).

The diversity of the TCR repertoire, which reflects the clonal composition of T cells may be a predictive marker of response to cancer immunotherapy^44,45^. Previous reports have shown that anti-CTLA-4 treatment leads to a significant broadening of CD8^+^ T-cell responses specific for melanoma and an increased diversity of T cells in blood after CTLA-4 blockade^46,47^.

In the present study, TCR repertoire analysis did not show any difference in TRB diversity or evenness in TILs cultured with or without anti-CTLA-4 antibody. Although we found that TIL subjected to CTLA-4 blockade did show a moderate increase in TRB richness, this, however, was not significant. It is highly likely that the overall sample size was too small to detect a statistically significant difference in these groups and further investigation is warranted to explore whether CTLA-4 blockade affects the clonality or diversity of TCR clones.

In conclusion, we show that targeting CTLA-4 within the initial tumor fragment cultures for ACT in ovarian cancer, increases TIL proliferation and favors the expansion of tumor-reactive CD8^+^ TILs. These results warrant clinical testing of the addition of an anti-CTLA-4 antibody during the initial TIL expansion phase to improve ACT using TILs in this hard-to-treat population.

## Material and Methods

### Patient material

We obtained metastatic tumor lesions from 14 individual patients with histologically verified ovarian cancer. Seven patients participated in a phase I clinical trial (Trial Number NCT02482090) and 6 patients in a phase I/II clinical trial (Trial Number NCT03287674) at CCIT, Herlev Hospital (Herlev, Denmark). Additionally, we obtained a metastatic lesion from one patient in a preclinical study to evaluate TIL therapy for ovarian cancer.

### Generation of TIL cultures

The majority of TIL cultures were established from freshly resected tumor lesions, which were immediately transported to the laboratory and cut into 1 to 3 mm^3^ fragments. For three patients, fragments cryopreserved in dimethyl sulfoxide (DMSO) were used to establish initial TIL cultures. Fragments were placed into individual wells of a 24-well plate (Nunc, 142475) with 2 ml of complete medium (CM) consisting of RPMI1640 with GlutaMAX, 25 mM HEPES pH 7.2 (Gibco, 72400–021), 10% heat-inactivated human AB serum (HS; Sigma-Aldrich, H4522–100ML), 100 U/ml penicillin, 100 μg⁄ml streptomycin (Gibco, 15140-122), 1.25 μg/ml Fungizone (Bristol-Myers Squibb, 49182) and 6,000 IU⁄ml Interleukin 2 (IL-2; Proleukin, Novartis, 004184). Where indicated, 5 μg/ml anti-CTLA-4 (Bristol Myers Squibb) was added to the initial TIL cultures. The 24-well plates were placed in a humidified 37 °C incubator with 5% CO_2_. Half of the medium was replaced three times per week. Initial TIL cultures were established *in vitro* according to the young TIL method by pooling TIL cultures derived from separate fragments^48^. TIL cultures were considered established when more than 2 × 10^6^ cells/fragment could be generated within 35 days in one of the two culture conditions. In order to limit differences in culture time of initial TILs under different conditions from the same patient, all initial TILs from one patient were harvested within three days. TILs were further expanded in a small-scale version of the 14-day rapid expansion protocol, as previously described^29^. Here, freshly generated TILs or cryopreserved TILs rested in CM for two days were used. Briefly, 1 × 10^5^ TIL, 2 × 10^7^ allogeneic feeder cells from healthy donors, 30 ng/ml OKT3 antibody (anti-CD3, Janssen-Cilag, 24639 G), master mix made of 50% CM and 50% rapid expansion medium (RM) consisting of AIM-V medium (Gibco, 12055-083) and 1.25 μg⁄ml Fungizone supplemented with 6,000 IU⁄ml IL-2 with 10% HS were mixed in T25 tissue culture flasks (Nunc, 156367) and cultured at 37 °C in 5% CO_2_. On day 0 and 7, 5 μg/ml anti-CTLA-4 was added to the respective cultures. The rapid expansion of each setup was performed in duplicates. Cells were split into larger flasks and additional media was added in order to keep a cell density between 1–2 × 10^6^ cells/ml. The expansion fold was determined on day 7, 9, 12 and 14 of the rapid expansion and TILs were harvested and cryopreserved on day 14 of the expansion.

### Tumor cell cultures

Tumor cell lines were established either directly from tumor fragments, from media used for transportation of the tumor specimen or from enzymatically digested fresh tumor fragments and validated by Cytospin centrifugation of the cell suspension for morphologic evaluation and formalin-fixed paraffin-embedded followed by immunohistochemistry staining for various ovarian cancer markers; CA125, EpCAM, PAX8, p16, p53, CK7, the mesothelial cell marker Calretinin and the proliferation marker Ki67. R10 medium consisting of RPMI1640 with GlutaMAX, 25 mM HEPES pH 7.2, 100 U⁄ml penicillin, 100 μg⁄ml streptomycin and 10% fetal bovine serum (FBS; Gibco, 10270–106) was used for culturing tumor cell lines.

### Phenotypic characterization of TIL

Staining of both initial and expanded TILs was performed on fresh cells or cells rested in RPMI1640 with GlutaMAX, 25 mM HEPES pH 7.2, 100 U⁄ml penicillin, 100 μg⁄ml streptomycin with 10% HS overnight. The cells were washed in phosphate-buffered saline (PBS, Lonza, BE176-512F), stained at 4 °C for 30 minutes, washed and resuspended in PBS. The fluorochrome-labeled monoclonal antibodies BV510 CD, PerCP CD4, BV421 CD8, FITC CD45RA, PE CD27, PE-Cy7 C-C chemokine receptor type 7 (CCR7), APC CD62L, FITC CD57, PE inducible T-cell co-stimulator (ICOS), PE-Cy7 CD69, APC CD28, FITC CD16, PE CD137, PE-Cy7 CD56, APC CTLA-4, FITC lymphocyte-activation gene 3 (LAG-3), PE B- and T-lymphocyte attenuator (BTLA), PE-Cy7 PD-1 and APC T-cell immunoglobulin and mucin-domain containing-3 (TIM-3) were used. The manufacturer information and clones are listed in Supplementary Table S3. The stained cells were analyzed with a FACS Canto II instrument (BD Biosciences). The gating strategy is shown in Supplementary Fig. S7.

### Evaluation of tumor reactivity

Anti-tumor reactivity of *in vitro* expanded TILs was evaluated after co-culture of the TILs with autologous tumor cell lines pretreated with 100 IU/ml IFN-γ (Peprotech, 300-02-100UG) in a ratio of 3:1 for 5 hours. Golgi plug (BD Biosciences, 51-2301KZ) and BV421 CD107a were added at the beginning of incubation and TILs were stained with Near-IR Live/Dead (Life Technologies, L10119) and for surface markers FITC CD3, PE CD56, QDot605 CD8 and PerCP CD4. Subsequently, the cells were fixed and permeabilized (eBioscience, 00-5123-43, 00-5223-56 and 00-8333-56) overnight and stained for intracellular cytokines APC TNF and PE-Cy7 IFN-γ. The manufacturer information and clones of the flow cytometry antibodies are listed in Supplementary Table S4. The TILs were analyzed with a FACS Canto II. Tumor-reactive TILs were defined as T cells expressing either TNF, IFN-γ or CD107a. The response in an unstimulated sample (negative control) was subtracted from the stimulated samples. The gating strategy is shown in Supplementary Fig. S8.

### RNA sequencing and TCR analysis

RNA sequencing (RNA-seq) was performed on initial TIL cultures with (n = 12) and without (n = 12) anti-CTLA-4 antibody supplement, and on REP cultures (n = 24) expanded from these initial TILs without the addition of anti-CTLA-4 (total n = 48) as previously described^49^. After demultiplexing, the sequences were analyzed using MiXCR^50,51^ version 3.0.3 (command: mixcr analyze shotgun; parameters:–species hs–starting-material rna–only-productive). VDJtools^52^ was applied to MiXCR output to derive TRB diversity, richness and evenness, using command CalcDiversityStats.

### Data and statistical analysis

Data analyses were carried out in Excel 2010 and Graphpad Prism 5. The change in the TIL expansion and phenotypic subpopulations was investigated for the statistical difference using Wilcoxon matched-pairs rank test. A two-sided p-value of <0.05 was considered statistically significant.

### Ethics approval

The Danish National Committee on Health Research Ethics approved the scientific use of the patient material.

### Accordance

The study was conducted in accordance with the Helsinki Declaration.

### Consent for publication

All patients included in the study have signed informed consent to participate.

## Supplementary information

Supplementary Information.

## Data Availability

Data and material generated and analyzed during the current study can be available upon reasonable request to the corresponding author.
