# Supplementary material for: CTLA-4 blockade boosts the expansion of tumor-reactive CD8+ tumor-infiltrating lymphocytes in ovarian cancer
Source: Sci Rep. 2020 Mar 3;10:3914. doi: 10.1038/s41598-020-60738-4 (PMC7054305; doi:10.1038/s41598-020-60738-4)
Supplement: Supplementary file 1 — Supplementary Information. [file 41598_2020_60738_MOESM1_ESM.pdf]

CTLA-4 blockade boosts the expansion of tumor-reactive CD8+ tumor-infiltrating lymphocytes in ovarian cancer

Authors:

Christina Friese[1], Katja Harbst[2], Troels Holz Borch[1], Marie Christine Wulff Westergaard[1], Magnus Pedersen[1], Anders Kverneland[1], Göran Jönsson[2], Marco Donia[1], Inge Marie Svane*[1], Özcan Met*[1,3]

Affiliations:

[1]Center for Cancer Immune Therapy (CCIT), Department of Oncology, Herlev Hospital, Herlev, Denmark.

[2]Department of Clinical Sciences Lund, Division of Oncology and Pathology, Lund University, Lund, Sweden

[3]Department of Immunology and Microbiology, Faculty of Health and Medical Sciences, University of Copenhagen, Denmark.

***These authors share senior authorship**

Corresponding Authors:

Özcan Met, Email: ozcan.met@regionh.dk, phone: +4538689229, fax: +4538684153

and Inge Marie Svane, Email: inge.marie.svane@regionh.dk, phone: +4538689339, fax: +4538684153

Supplementary Information

Supplementary Table S1. CD8$^+$ T cells in the initial and expanded TIL cultures.

Patient	Before expansion		After expansion			
α-CTLA-4	-	+	-/-	-/+	+/-	+/+
GY1508.05	18.7%	96.3%	47.2%	45.7%	97.5%	97.0%
GY1508.06	25.4%	77.9%	9.7%	9.5%	88.6%	88.7%
GY1508.07	22.6%	78.1%	2.0%	1.0%	19.3%	17.3%
GY1508.08	2.9%	38.1%	1.3%	3.2%	14.1%	11.8%
GY1508.09	83.9%	77.0%	58.2%	60.4%	40.5%	57.2%
GY1508.10	4.2%	5.5%	1.9%	0.8%	2.0%	3.2%
GY1508.11	12.9%	43.4%	4.9%	3.4%	53.5%	56.2%
EOC.TIL.27	0.3%	15.5%	0.1%	0.1%	71.3%	69.6%
GY1721.01	26.5%	28.2%	23.9%	21.8%	32.7%	32.5%
GY1721.02	8.4%	22.7%	12.8%	2.5%	0.9%	1.0%
GY1721.03	63.7%	48.3%	71.7%	68.8%	43.3%	43.8%
GY1721.04	14.5%	3.8%	32.2%	29.4%	3.6%	3.8%
GY1721.05	39.7%	41.8%	57.7%	55.9%	39.9%	40.8%
GY1721.07	15.2%	0.0%	0.9%	0.5%	0.2%	0.0%

Supplementary Table S2. CD4$^+$ T cells in the initial and expanded TIL cultures.

Patient	Before expansion		After expansion			
α-CTLA-4	-	+	-/-	-/+	+/-	+/+
GY1508.05	76.8%	1.3%	51.6%	53.1%	1.9%	2.1%
GY1508.06	71.5%	11.5%	79.8%	86.5%	8.7%	8.4%
GY1508.07	72.9%	18.9%	96.1%	97.5%	77.5%	79.3%
GY1508.08	72.3%	60.6%	96.9%	95.1%	49.6%	55.1%
GY1508.09	10.7%	22.1%	13.5%	11.0%	40.2%	28.8%
GY1508.10	93.6%	86.5%	96.6%	98.2%	94.4%	92.1%
GY1508.11	82.9%	48.0%	92.6%	94.8%	29.3%	32.3%
EOC.TIL.27	99.3%	83.2%	97.1%	97.5%	26.4%	28.8%
GY1721.01	54.0%	62.4%	74.3%	76.2%	62.9%	63.1%
GY1721.02	86.1%	55.8%	77.5%	92.5%	96.9%	96.8%
GY1721.03	26.0%	44.7%	25.9%	28.3%	50.8%	49.1%
GY1721.04	84.9%	95.2%	66.7%	69.6%	95.9%	94.7%
GY1721.05	55.7%	45.9%	33.5%	37.5%	55.3%	54.6%
GY1721.07	25.5%	96.2%	96.4%	96.6%	96.3%	98.5%

Supplementary Table S3. Flow cytometry antibodies used for phenotyping.

Antigen	Fluorochrome	Clone	Manufacturer	Catalog No.
**BTLA**	PE	J168-50	BD Biosciences	558485
**CCR7**	PE-Cy7	3D12	BD Biosciences	557648
**CD3**	BV510	SK7	BD Biosciences	740202
**CD4**	PerCP	SK3	BD Biosciences	345770
**CD8**	BV421	RPA-T8	BD Biosciences	562428
**CD16**	FITC	DJ130c	Dako	F7011
**CD27**	PE	L128	BD Biosciences	340425
**CD28**	APC	CD28.2	BD Biosciences	559770
**CD45RA**	FITC	L48	BD Biosciences	335039
**CD56**	PE-Cy7	B159	BD Biosciences	557747
**CD57**	FITC	NK-1	BD Biosciences	555619
**CD62L**	APC	DREG-56	BD Biosciences	559772
**CD69**	PE-Cy7	FN50	BD Biosciences	557745
**CD137**	PE	4B4-1	BD Biosciences	555956
**CTLA-4**	APC	L3D10	BioLegend	349908
**ICOS**	PE	DX29	BD Biosciences	557802
**LAG-3**	FITC	17B4	LS Bioscience	LS-B2237
**PD-1**	PE-Cy7	EH12.1	BD Biosciences	561272
**TIM-3**	APC	F38-2E2	eBioscience	17-3109-42

Supplementary Table S4. Flow cytometry antibodies used for intracellular cytokine staining.

Antigen	Fluorochrome	Clone	Manufacturer	Catalog No.
**CD3**	FITC	SK7	BD Biosciences	345763
**CD4**	PerCP	OKT4	BioLegend	317432
**CD8**	QDot605	3B5	Invitrogen	Q10009
**CD56**	PE	NCAM16.2	BD Biosciences	345812
**CD107a**	BV421	H4A3	BD Biosciences	562623
**IFN-γ**	PE-Cy7	B27	BD Biosciences	557643
**TNF**	APC	Mab11	BD Biosciences	554514

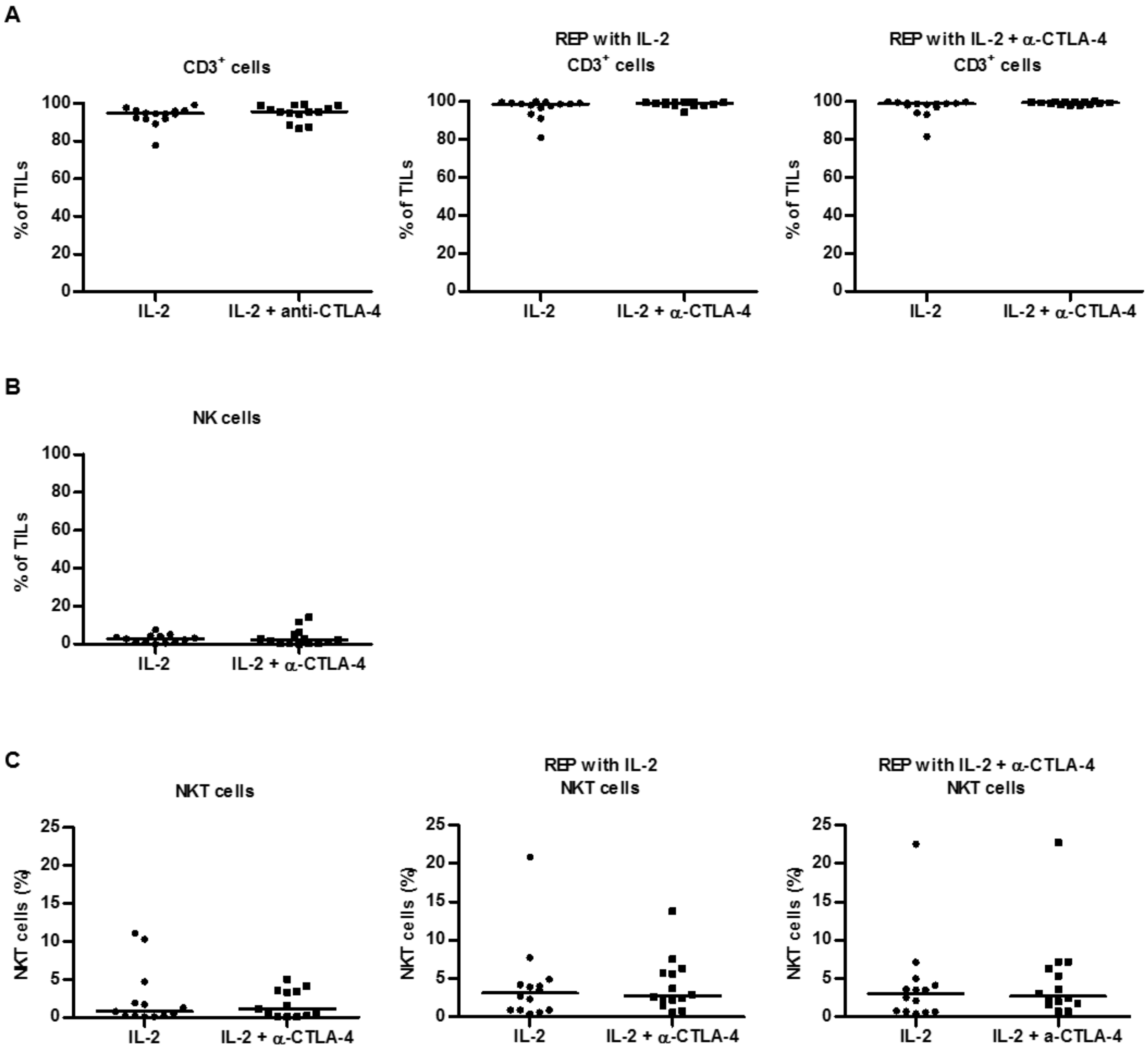

<u>Supplementary Figure S1. CD3$^+$ T and NK cells.</u>

(A) Scatterplots showing the proportion of CD3$^+$ cells in initial TIL cultures, TILs expanded in high-dose IL-2 only and TILs expanded in high-dose IL-2 and anti-CTLA-4. The legend indicates the culture conditions during the initial TIL expansion. Data are presented with median.

(B) Scatterplot showing the proportion of NK cells ($CD3^{-}CD56^{+}$) in initial TIL. Data are presented with median.

(C) Scatterplots showing the proportion of NKT cells ($CD3^{+}CD56^{+}$) in initial TIL cultures, TILs expanded in high-dose IL-2 only and TILs expended in high-dose IL-2 and anti-CTLA-4. The legend indicates the culture conditions during the initial TIL expansion. Data are presented with median.

Supplementary Figure S2

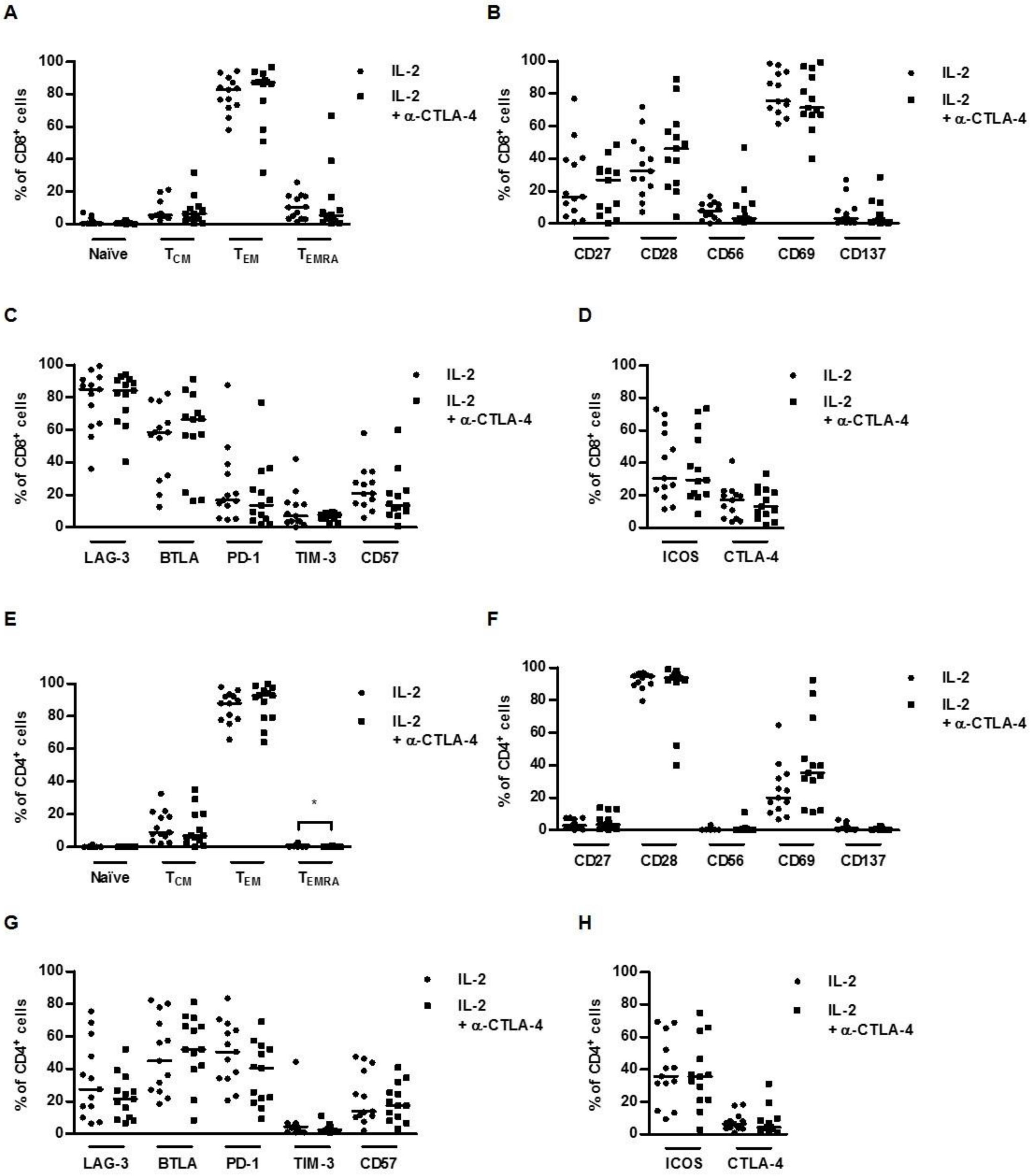

Supplementary Figure S2. Phenotype of initial TILs.

(A and E) Scatterplot showing the percentages of naïve (T_N), central memory (T_{CM}), effector memory (T_{EM}) and T_{EMRA}, **(B and F)** scatterplot showing the percentages of $CD27^+$, $CD28^+$, $CD56^+$, $CD69^+$ and $CD137^+$ cells, **(C and G)** scatterplot showing the percentages of LAG-3^+, $BTLA^+$, PD-1^+, TIM-3^+ and $CD57^+$ cells and **(D and H)** scatterplot showing percentages of $ICOS^+$ and CTLA-4^+ cells in **(A-D)** the $CD8^+$ T-cell population and in **(E-H)** the $CD4^+$ T-cell population in initial TIL culture supplemented with anti-CTLA-4 antibody and non-supplemented initial TILs (n=13). The legend indicates the culture conditions during the initial TIL expansion. T_N, T_{CM}, T_{EM} and T_{EMRA} cells were defined as $CD45RA^+$ $CD62L^+$, $CD45RA^-$ $CD62L^+$, $CD45RA^-$ $CD62L^-$ and $CD45RA^+$ $CD62L^-$, respectively. Data are presented with median.

The different conditions were compared using Wilcoxon signed rank test. Statistically significant differences are indicated with *, ** or *** for p-values less than 0.05, 0.01 or 0.001, respectively.

Supplementary Figure S3

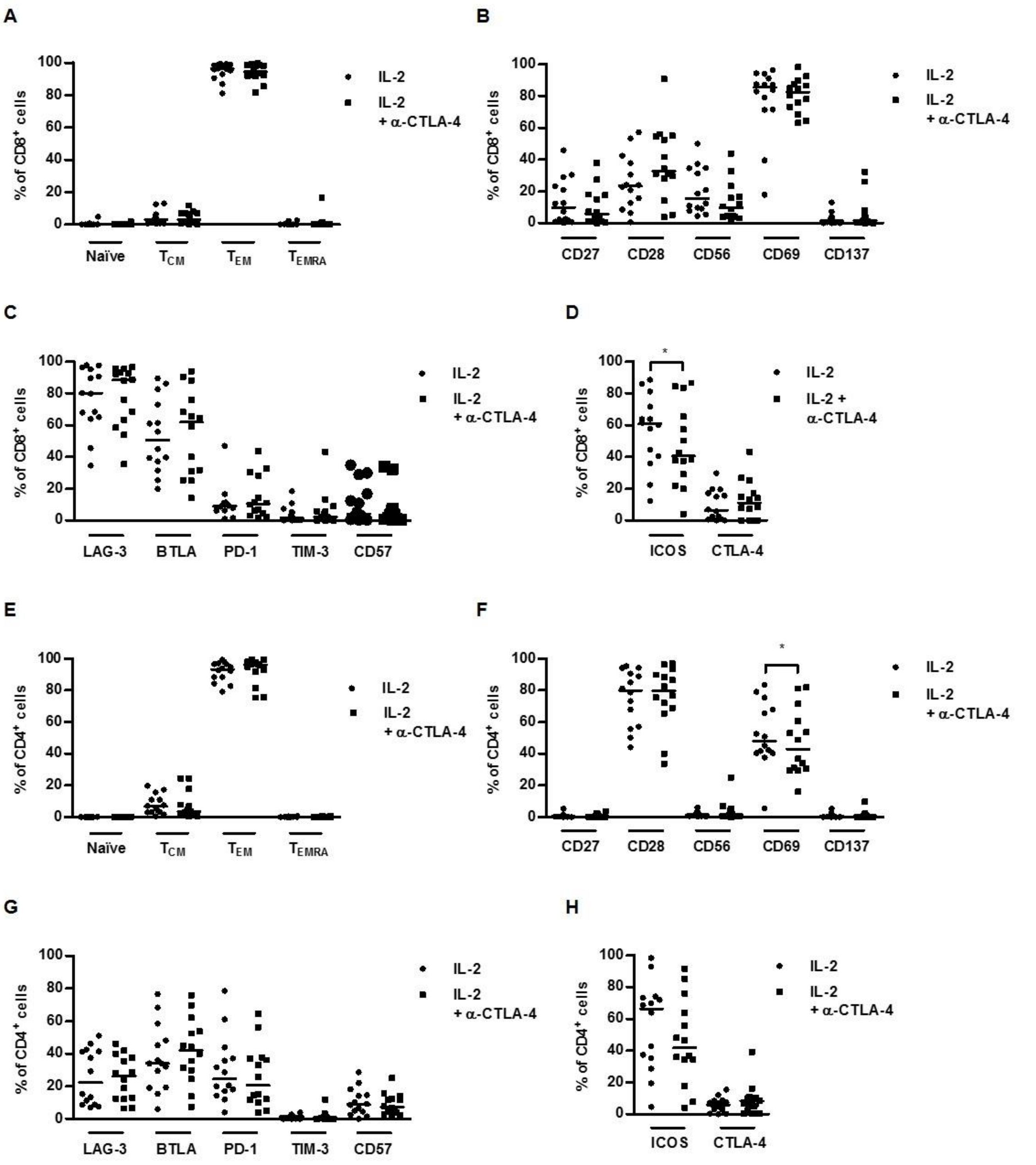

Supplementary Figure S3. Phenotype of expanded TILs.

(A and E) Scatterplot showing the percentages of naïve (T_N), central memory (T_{CM}), effector memory (T_{EM}) and T_{EMRA}, **(B and F)** scatterplot showing the percentages of $CD27^+$, $CD28^+$, $CD56^+$, $CD69^+$ and $CD137^+$ cells, **(C and G)** scatterplot showing the percentages of exhaustion markers LAG-3, BTLA, PD-1, TIM-3 and CD57 and **(D and H)** scatterplot showing percentages of $ICOS^+$ and $CTLA^+$ cells in **(A-D)** the $CD8^+$ T-cell population and in **(E-H)** the $CD4^+$ T-cell population in TILs expanded in high-dose IL-2. The legend indicates the culture conditions during the initial TIL expansion. T_N, T_{CM}, T_{EM} and T_{EMRA} cells were defined as $CD45RA^+$ $CD62L^+$, $CD45RA^-$ $CD62L^+$, $CD45RA^-$ $CD62L^-$ and $CD45RA^+$ $CD62L^-$, respectively. Data are presented with median.

The different conditions were compared using Wilcoxon signed rank test. Statistically significant differences are indicated with *, ** or *** for p-values less than 0.05, 0.01 or 0.001, respectively.

Supplementary Figure S4

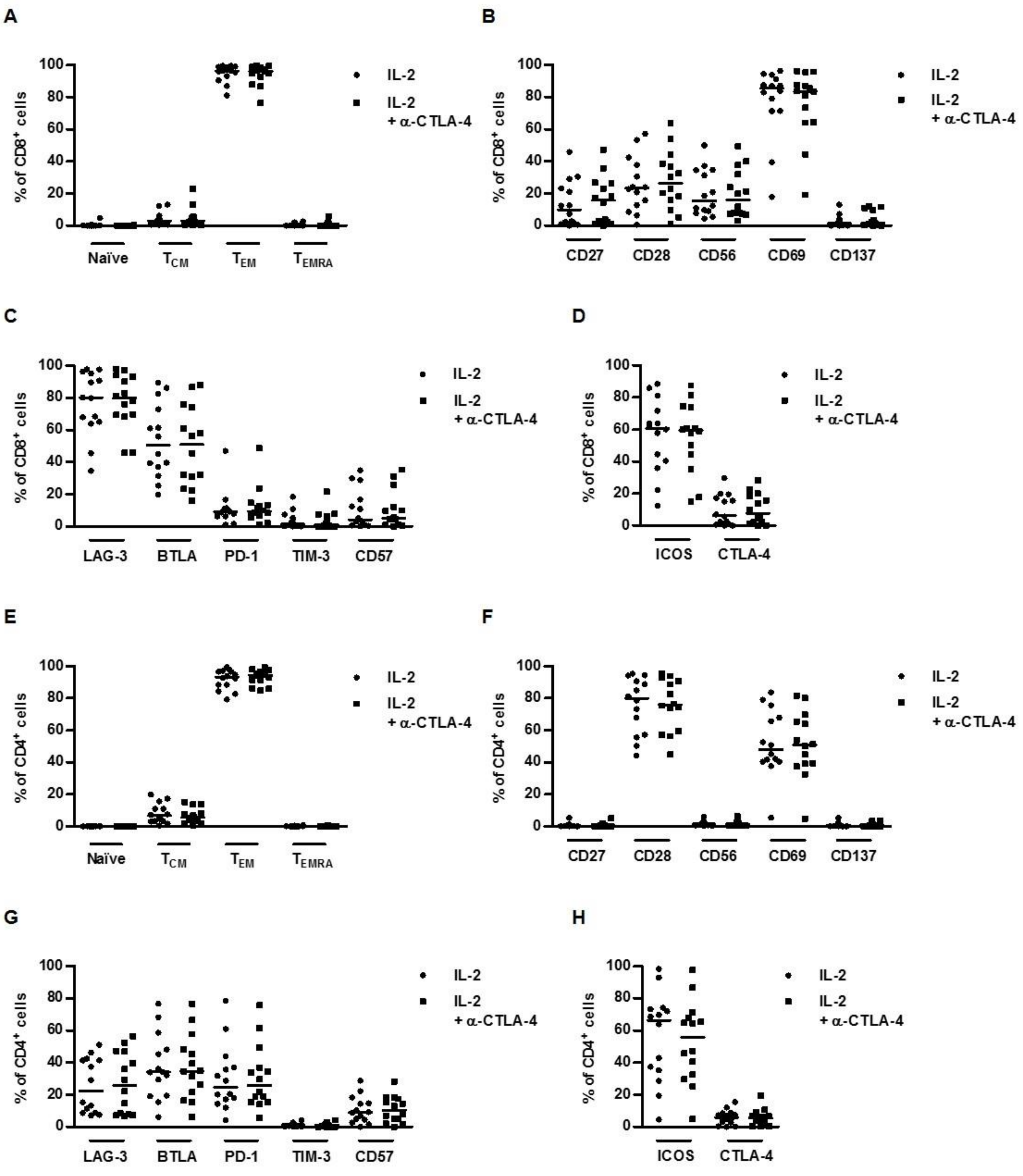

Supplementary Figure S4. Phenotype of TILs expanded from TILs not supplemented with anti-CTLA-4 antibody.

(A and E) Scatterplot showing the percentages of naïve (T_N), central memory (T_{CM}), effector memory (T_{EM}) and T_{EMRA}, **(B and F)** scatterplot showing the percentages of $CD27^+$, $CD28^+$, $CD56^+$, $CD69^+$ and $CD137^+$ cells, **(C and G)** scatterplot showing the percentages of exhaustion markers LAG-3, BTLA, PD-1, TIM-3 and CD57 and **(D and H)** scatterplot showing percentages of $ICOS^+$ and $CTLA^+$ cells in **(A-D)** the $CD8^+$ T-cell population and in **(E-H)** the $CD4^+$ T-cell population in TILs expanded from initial TILs not supplemented with anti-CTLA-4. The legend indicates the culture conditions during the rapid TIL expansion. Data are presented with median.

Supplementary Figure S5

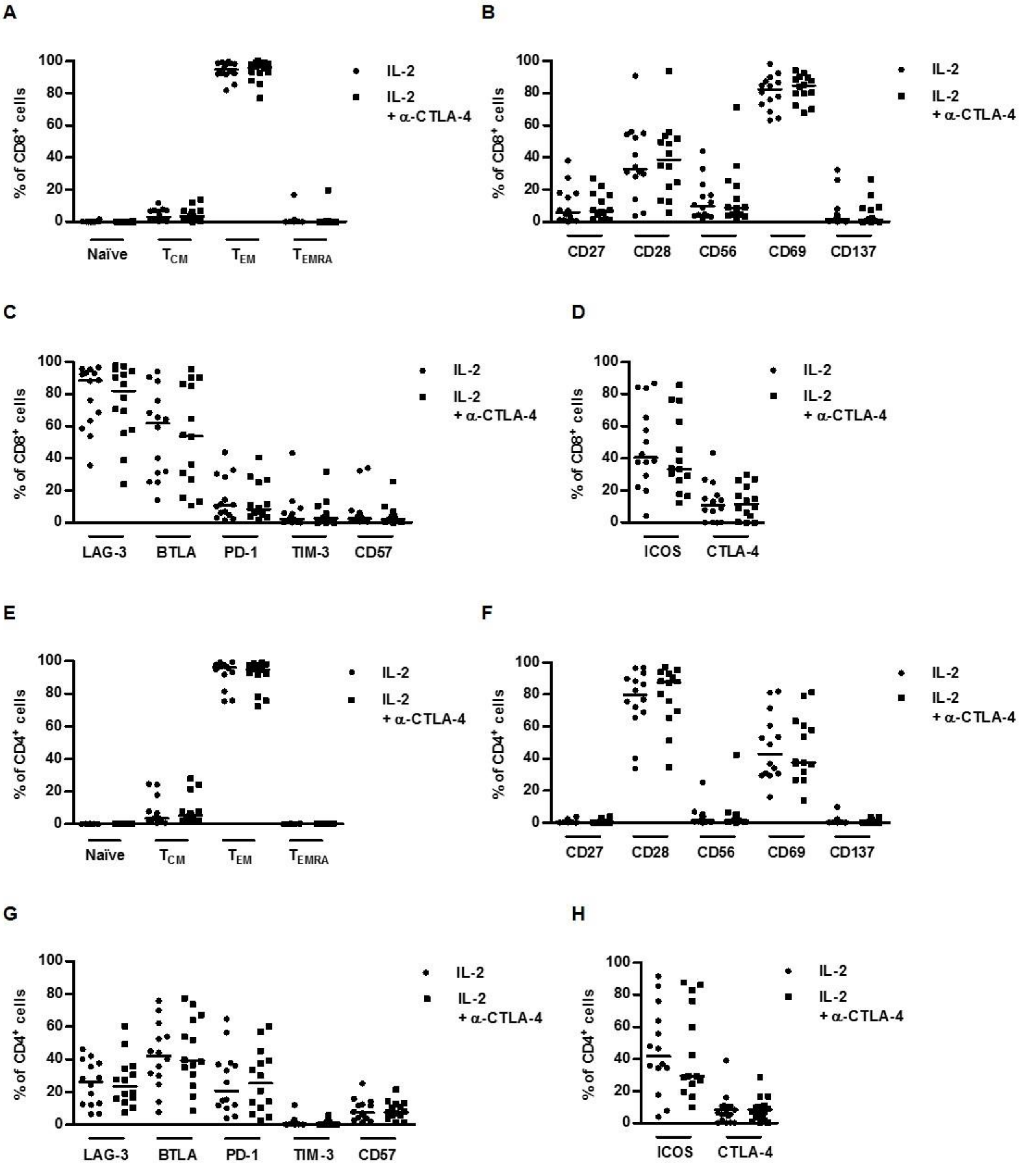

Supplementary Figure S5. Phenotype of TILs expanded from TILs supplemented with anti-CTLA-4 antibody.

(A and E) Scatterplot showing the percentages of naïve (T_N), central memory (T_{CM}), effector memory (T_{EM}) and T_{EMRA}, **(B and F)** scatterplot showing the percentages of $CD27^+$, $CD28^+$, $CD56^+$, $CD69^+$ and $CD137^+$ cells, **(C and G)** scatterplot showing the percentages of LAG-3^+, $BTLA^+$, PD-1^+, TIM-3^+ and $CD57^+$ cells and **(D and H)** scatterplot showing percentages of $ICOS^+$ and CTLA-4^+ cells in **(A-D)** the $CD8^+$ T-cell population and in **(E-H)** the $CD4^+$ T-cell population in TILs expanded from initial TILs supplemented with anti-CTLA-4. The legend indicates the culture conditions during the rapid TIL expansion. Data are presented with median.

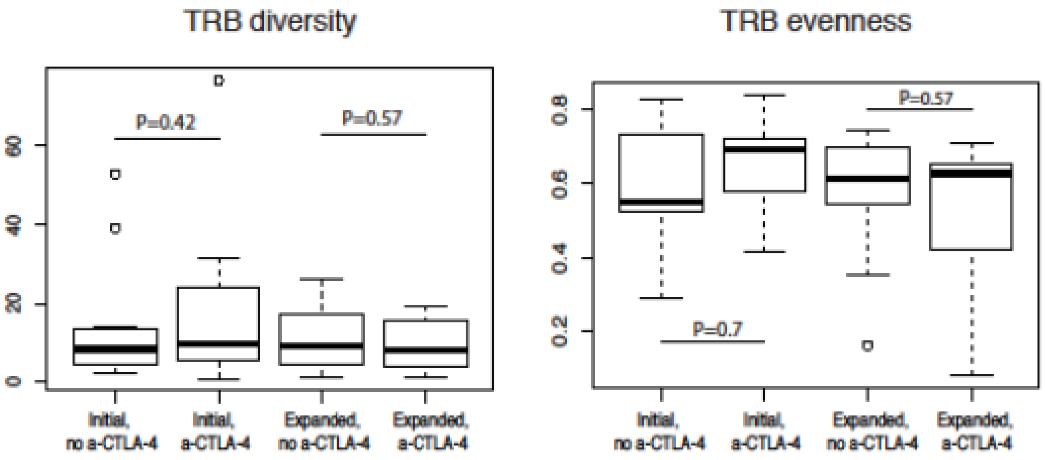

Supplementary Figure S6. TRB diversity parameters of initial and expanded TILs.

TRB diversity (left) and evenness (right) in the initial and expanded cultures with and without anti-CTLA-4 antibody, as derived by TCR repertoire analysis.

Supplementary Figure S7

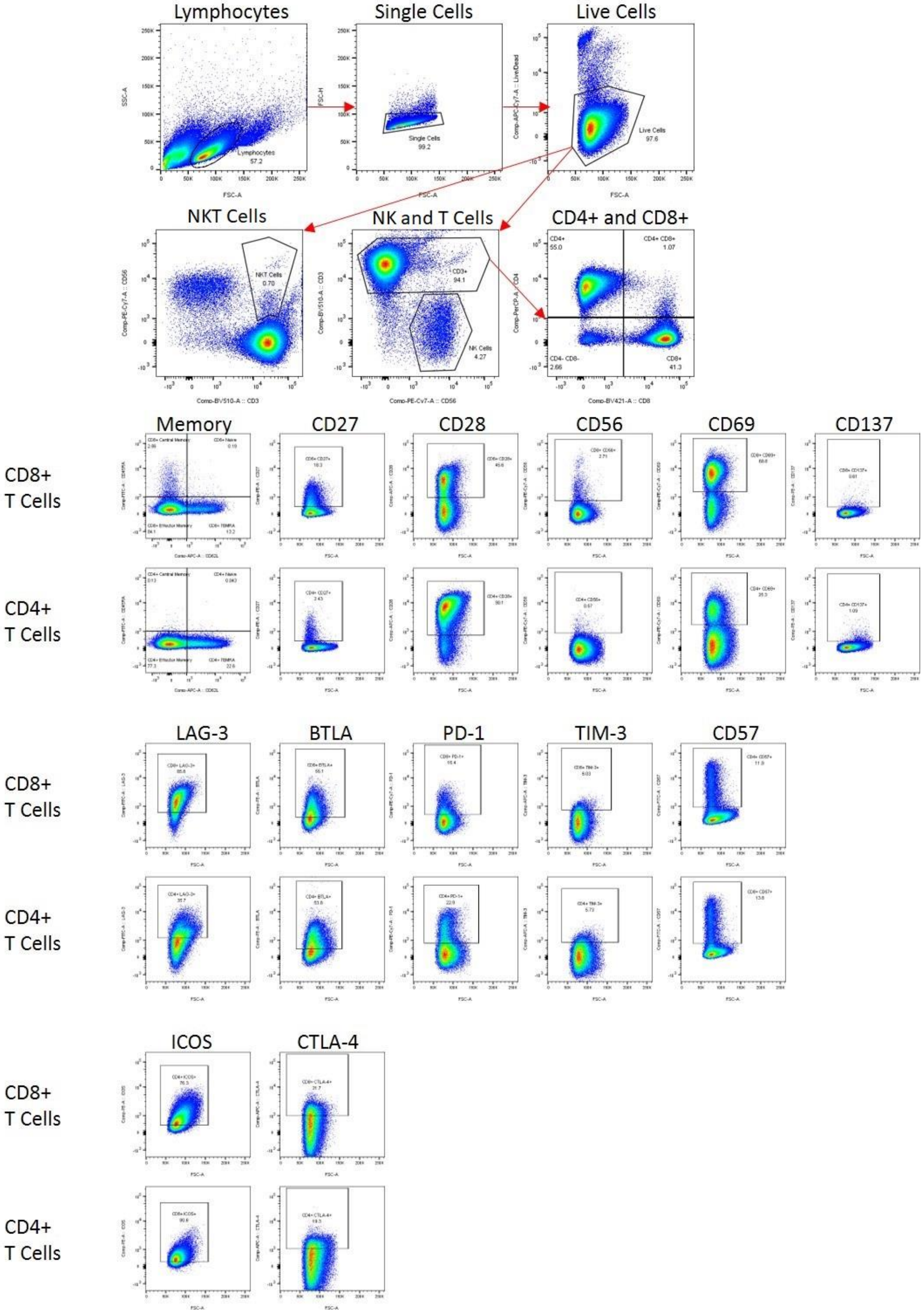

Supplementary Figure S7. Gating Strategy of the phenotyping of TILs.

FACS plots illustrating the gating strategy used for the phenotype analysis of both initial and expanded TIL cultures. The FACS data from the unsupplemented initial TIL culture from patient GY1721.05 are shown. Lymphocytes, single cells, live cells, $CD3^+$, NK and NKT cells were gated consecutively. Subsequently, $CD4^+$ and $CD8^+$ cells within the $CD3^+$ cells and cells expressing activation and exhaustion markers were gated separately in the $CD4^+$ and $CD8^+$ cell populations.

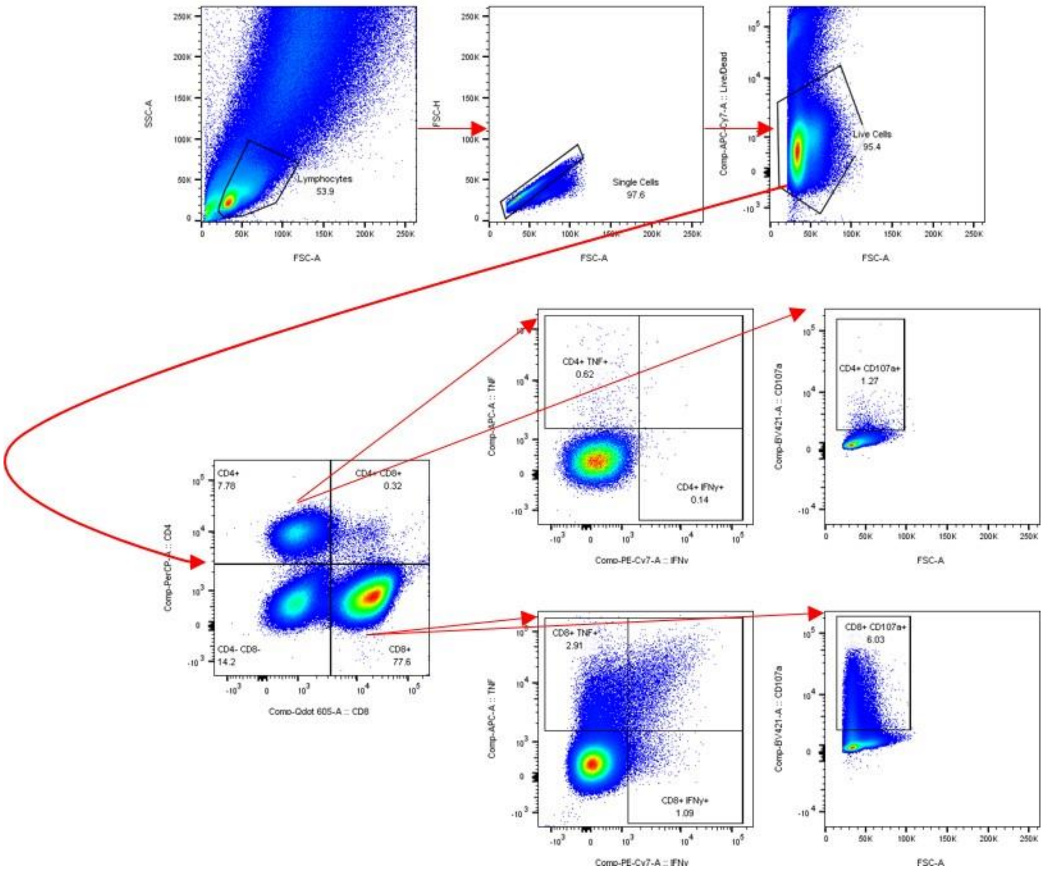

<u>Supplementary Figure S8. Gating strategy of the tumor reactivity analysis of TILs.</u>

FACS plots illustrating the gating strategy used for the functionality analysis of both initial and expanded TIL cultures after co-culture with autologous tumor cell lines. The FACS data from the TIL culture expanded from the initial TIL culture supplemented with anti-CTLA-4 antibody from patient GY1508.06 are shown. Lymphocytes, single cells, live cells and $CD4^+$ and $CD8^+$ cells were gated. Subsequently, cells secreting the cytokines TNF and IFNγ (left panels) or expressing CD107a (right panels) in $CD4^+$ cells (upper panels) and $CD8^+$ cells (lower panels) were gated.
